# Mechanical and Fracture Properties of Long Fiber Reinforced Geopolymer Composites

**DOI:** 10.3390/ma14185183

**Published:** 2021-09-09

**Authors:** Kinga Korniejenko, Beata Figiela, Krzysztof Miernik, Celina Ziejewska, Joanna Marczyk, Marek Hebda, An Cheng, Wei-Ting Lin

**Affiliations:** 1 Faculty of Materials Engineering and Physics, Cracow University of Technology, al. Jana Pawła II 37, 31-864 Kraków, Poland; krzysztof.miernik@pk.edu.pl (K.M.); celina.ziejewska@pk.edu.pl (C.Z.); joanna.marczyk@pk.edu.pl (J.M.); mhebda@pk.edu.pl (M.H.); 2 Department of Civil Engineering, National Ilan University, No. 1, Sec. 1, Shennong Rd., Yilan City 26041, Taiwan; ancheng@niu.edu.tw (A.C.); wtlin@niu.edu.tw (W.-T.L.)

**Keywords:** geopolymer composite, fiber reinforcement, additive technology, long fiber

## Abstract

The aim of the article is to analyze the structure and mechanical properties in terms of the cracking mechanics of geopolymer composites based on fly ash and river sand, as well as metakaolin and river sand with three types of reinforcement material: glass fiber, carbon fiber, and aramid fiber, in terms of their use in additive manufacturing. Geopolymer composites were reinforced with fibers in a volume ratio of 0.5%, 1.0%, and 2.0%. Subsequently, these samples were subjected to bending strength tests in accordance with the European standard EN 12390-3. The addition of fibers significantly improved the bending strength of all composites made of metakaolin and sand. The reinforcement with aramid fiber in the amount of 2.0% resulted in more than a 3-fold increase in strength compared to the reinforcement-free composites. An analysis of the morphology of the fibers was carried out on the basis of photos taken from an electron microscope. The correct addition of fibers changes the nature of the fracture from brittle to more ductile and reduces the number of cracks in the material.

## 1. Introduction

With the development of modern civilization, man began to emit increasing amounts of greenhouse gases—mainly carbon dioxide (CO_2_)—which enter the atmosphere. The increase in the concentration of greenhouse gases leads to an increase in the average temperature of the Earth’s surface, which leads to negative consequences for both nature and man [1,2]. A significant proportion of the CO_2_ emitted to the atmosphere is bound with the cement industry. Carbon dioxide in the cement production process is emitted as a result of: consumption of fossil fuels directly in the burning process (approx. 40%), transport of raw materials (approx. 5%), combustion of fossil fuels needed to generate electricity used in a cement plant (approx. 5%), and the decarbonization process (approx. 50%) [3]. It is estimated that the production of 1000 kg of ordinary Portland cement (OPC) emits almost 900 kg of CO_2_ into the atmosphere, which, according to data from 2018, is responsible for about 3.5% of its global, anthropogenic emission [3]. For this reason, science is looking for alternatives to Portland cement that will reduce the environmental impact of the cement industry. One of them may be geopolymers. The production of geopolymers releases much smaller amounts of CO_2_ into the atmosphere, and it can be a real alternative to waste management from the energy or metallurgical sector (for example, fly ash, slag) or also other sectors such as the construction industry (for example, construction and demolition waste) or mining (for example, different mine tailings) [4,5,6,7].

Statistics show that the problem of CO_2_ emission will be increasing during the next few years. It is connected, i.e., with the world cement production that will increase, from around 4.3 billion tonnes in 2015 to around 6.1 billion tonnes in 2050 [8]. The world leader in cement production is China, which is rapidly urbanizing, where more than half of its global production comes from [8]. In addition, OPC production, in addition to the emission of large amounts of carbon dioxide and energy consumption (approximately 4.8 GJ/t), is threatened with the depletion of limestone reserves [8]. Therefore, there is a need to look for environmentally friendly alternatives to OPC. One of them may be geopolymeric materials, which have attracted attention due to their excellent mechanical and chemical properties, analogous to those offered by OPC. Moreover, it is estimated that, compared to the production of OPC, the synthesis of geopolymers is much less energy-consuming and emits about 5–6 times less CO_2_ than the production of Portland cement [9].

Geopolymers are inorganic polymers resulting from the synthesis of silicon and aluminum obtained geologically from minerals [10,11] at a temperature not exceeding 100 °C [12,13]. The terms geopolymers and alkaline activated materials are used in the literature very often interchangeably and treated equally by many authors [14,15]. This approach appears to be incorrect [16,17], due to differences in the structure of these materials [16]. Justification is related to the structure differences that are evident in NMR microstructure studies [16]. Taking into account the entire geopolymerization, alkaline activation is only the first step in the creation of geopolymeric materials [16]. Alkaline activated materials do not form lattice, but only a two-dimensional structure, which in turn affects their properties. Due to the different structure, geopolymeric and alkaline activated materials may have different physicochemical and functional properties, in particular resistance to chemical agents, functional properties, fire resistance and long-term properties [16]. It should be noted that the mechanical properties of alkaline activated materials may be even higher than that of geopolymers in the short term [16]. The raw material from which it will be made and additives in the material synthesis process will have the strongest influence on the process of creating the material [17]. In particular, the high aluminum content and the low calcium content favor the formation of geopolymeric materials. It should be noted that the sources of the literature do not specify the exact values of individual elements or their oxides in the base material [16].

The materials most commonly used for the production of geopolymers are metakaolin [18], pyroclastic volcanic tuffs, fly ash, or slags [19,20], thus both substances of natural origin and secondary raw materials [21]. Geopolymers are made by mixing aluminosilicates with an aqueous solution of sodium silicate and usually a strong sodium or potassium base [22,23]. In addition, the new solutions based on alkali can be prepared using mixtures of one part, such as: anhydrous sodium metasilicate (solid activator) [24,25,26]. One-part mixtures usually have a much lower carbon footprint than two-part-based mixtures [24], but also often lower mechanical properties [25,26]. Much less often used are the other activators for geopolymer materials such as phosphate acid or lithium silicate [24,25,26,27].

The attractiveness of geopolymers lies in their friendly impact on the environment due to the precursors from which they can be produced and the characteristic properties of finished products, such as immobilization of toxic substances, binding of heavy metal elements [28], fire resistance and refractoriness [29,30], as well as high strength properties equal to the strength of Portland cement [31]. These materials can successfully replace conventional building materials in specialist applications [31,32]. The addition of various types of fiber to the geopolymer matrix reduces the propagation of cracks in the material and transfers loads [33]. The geopolymers has also their limitations, including: lacks of regulations for application in building industry in many countries, changeable process of activators, problems in process repetitiveness, effloresces that could affect materials’ durability, and others [34,35].

Currently, one of the limitations of the widespread use of geopolymeric materials is their relatively low fracture toughness [36]. This limits the use of geopolymeric materials in many areas, including construction. An innovation in the production of geopolymers and thus the obtaining of various shapes is the use of 3D printing to print geopolymer mortar [37,38,39,40]. The problem with the production of these materials using additive technologies is due to the discontinuity of the material and the low tensile strength [41] and its damage can significantly reduce the dimensions of the elements produced for the construction industry [42]. Today, one of the most important research areas is the improvement bending strength of composites that would change their brittle nature of the breakthrough into ductile [43]. Currently, research is being conducted in the world on the possibility of introducing fibers as a filler into the geopolymer matrix [44,45,46]. These tests cover both continuous (long) fibers and short fibers [47,48,49,50,51]. Both types of fibers are also possible to use in additive manufacturing. Research shows that the addition of fibers improves the flexural strength and fracture toughness of geopolymeric materials. Fibers can also increase the amount of energy absorbed by the geopolymer before damage occurs. In particular, promising effects are obtained with the use of artificial fibers, that is, propylene fiber, glass fiber, carbon fiber, or basalt fiber [50,51,52,53,54,55,56].

Nematollahi et al. [51] investigated the influence of polypropylene fibers on the properties of geopolymeric material made in 3D printing technology. The addition of fibers in the amount of 0.5% improved the bending strength, but 0.75% and 1.0% of fibers increased the fracture energy of 3D-printed geopolymer samples while reducing the bending strength [51].

The use of fibers as reinforcement in geopolymer composites is considered a solution to improve its ductility, tensile strength, and compressive strength. Mishra and Panigrahi [57] conducted a review of solutions on this topic, which led to conclusions about the positive effect of fibers in shaping the desired properties [57].

In their research, Hambach and Volkmer [58] dealt with 3D printing of a paste of Portland cement fiber reinforced with a length of 3–6 mm: glass fibers, carbon fibers, and basalt fibers. The result was the production of new materials with high flexural strength up to 30 MPa and compression up to 80 MPa with 1% carbon fiber reinforcement [58].

Research on the use of aramid fiber as a reinforcement in geopolymer material is a modification of the research carried out to date [59]. Aramid fiber has a number of characteristic properties that make it attractive for applications as reinforcements in geopolymeric materials. Aramid fiber has a high tensile strength from 2400 N/mm^2^ to 3600 N/mm^2^ [60], abrasion and corrosion resistance [61], and high modulus of elasticity [62]. So far, research on the use of aramid fibers has been carried out with the use of a concrete matrix.

Manoj and Premkumar [63] conducted research on concrete reinforcement with aramid fibers, obtaining higher values of flexural strength (7.5 MPa to 8.23 MPa) and compressive strength (107.3 MPa to 116.1 MPa) compared to conventional concrete at ambient temperature [63].

The motivation for undertaking research was the literature analysis in the field of 3D printing of fiber-reinforced Portland cement paste. The analysis showed the necessity of works in material strengthening and obtaining high bending and compressive strength properties, as well as changing the fracture from brittle to more ductile [39,59,64,65,66]. Both metakaolin- and fly ash–based geopolymers were reinforced by long glass, carbon fiber, and aramid fibers. Currently, only a few types of such reinforcement have been investigated taking into account potential applications in 3D printing [67,68]. The article presented the novel knowledge based on research in this area.

The aim of the research carried out was to analyze the structure and mechanical properties in terms of geopolymer-based cracking mechanics of composites based on geopolymers depending on the amount of reinforcement material in terms of their application to additive manufacturing. Geopolymer composites were reinforced with long fibers: glass fiber, carbon fiber, and aramid fiber, and their bending strength was investigated. Moreover, the morphology of the fibers was performed on the basis of photos taken from an electron scanning microscope.

## 2. Materials and Methods

Samples were made with the use of two base materials: metakaolin and fly ash from the Skawina Combined Heat and Power Plant. A fine-grained aggregate was added to the base materials, river sand, in a 1:1 ratio. Sodium hydroxide (NaOH) solution containing the addition of sodium water glass was added to the mixture thus prepared. The sodium base solution was obtained from technical sodium hydroxide flakes combined with an aqueous solution of sodium silicate (type R-145, density 1.45 g/cm^3^) (ratio of sodium base to water glass: 1:2.5). Tap water was used to prepare the solution. The chemical composition and physical parameters of the water used are presented in Table 1 [69].

The resulting solution was thoroughly mixed and allowed to equilibrate to a constant concentration and temperature before combining with the solids of the mixture (24 h). The dry ingredients were mixed for 15 min in a low-speed mixer, and then the obtained masses were transferred to a set of prismatic forms, combined with the fiber roving. The samples were made with three types of long fibers (aramid, glass, and carbon) with their different percentages (0.5%, 1.0%, and 2.0% of the mass of loose components), and reference samples were made without the addition of fibers. The percentage selection of fibers was based on the literature review on the subject presented in the introduction [50]. The prepared masses in molds were compacted on a vibrating table. The samples were then cured for 24 h at a temperature of 75 °C in a laboratory dryer to receive a reasonable mechanical properties. After this time, the samples were cooled to ambient temperature, disassembled, and stored for the 90 days (the time used for full maturation of composites based on traditional cements). Seasoning was carried out under laboratory conditions, after which geopolymer composites were tested for bending strength.

Table 2 presents the compositions of the prepared samples, the number of series, and their mass ratios.

Mechanical strength tests and density determination were carried out for the produced sets of samples. The geometric method was used as a method for determining the density of composite geopolymer materials. Each sample was weighed with an electronic caliper (OVIBELL GmbH & Co. KG, Mülheim an der Ruhr, Germany) with a dimensional accuracy of 0.01 mm and weighed on a RADWAG PS200/2000R2 analytical balance (RADWAG Wagi Elektroniczne, Radom, Poland) with an accuracy of 0.0001/0.01 g. The density was determined on the basis of the average measurements from two samples.

The three-point bending strength tests were carried out in accordance with the EN 12390-3 standard: “Concrete tests-part 5: Bending strength” on the MATEST 3000 kN device—hydraulic press (Matest, Treviolo, Italy) at a speed of 0.05 MPa, on prismatic samples with dimensions of 50 mm × 50 mm × 200 mm. The distance between the support points was l = 150 mm. The tests were carried out on the standards for testing concrete mixes due to the similar nature of the material and the planned applications in the construction industry. Currently, no standards have been developed dedicated to the testing of geopolymeric materials.

The last step of the research was the assessment of the morphology of the samples, analyzed on the material remaining after the strength tests, bending strength tests (samples from composites), and on materials as delivered (original fibers, which were used to compare the degree of degradation). A JEOL JSM 5510LV scanning electron microscope (IXR Inc., Austin, TX, USA) was used for the research. Samples were prepared in advance. Small amounts of the materials were dried to constant weight and then placed on a carbonaceous support to drain the sample charge. The materials were sprayed with a thin layer of gold with JEOL–JEE-4X (IXR Inc., Austin, TX, USA). Observations were made at different magnifications.

## 3. Results

### 3.1. Density

**Figure 1:**
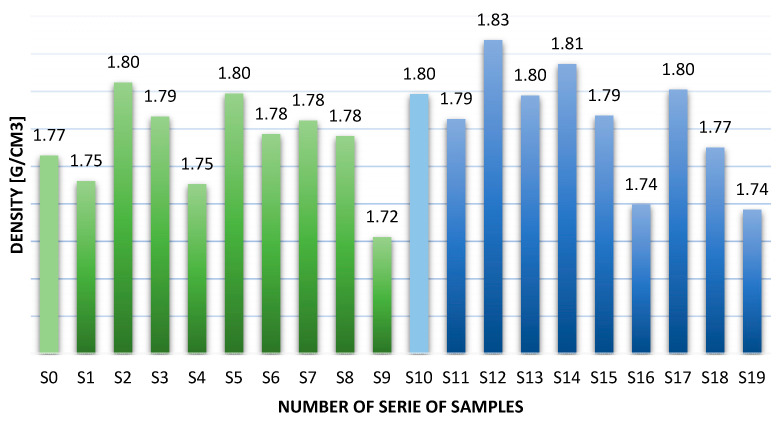
Density test result.

Figure 1 shows the results of the density determination for geopolymer composites.

The addition of fibers did not significantly affect the density of the materials. The addition of aramid fibers to the samples based on metakaolin and sand decreased their density as the amount of fibers increased. This is due to the low intrinsic density of aramid fibers [42], which are approximately 20% lighter than carbon fibers.

### 3.2. Flexural Strength Test

**Figure 2:**
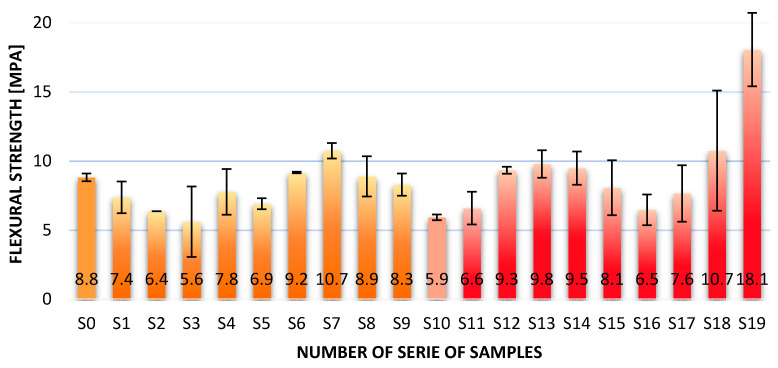
Results of the flexural strength test for geopolymer composites.

Figure 2 shows the results for the flexural strength test.

Measurements of the bending strength in the case of composites consisting of metakaolin and sand, in each variant of the addition of fibers, show the increase in strength in relation to the samples without additives. The measurements presented show that the highest values were obtained for the metakaolin-based composites with the addition of aramid roving. The highest bending strength is demonstrated by composites with 2% aramid fibers—18.1 MPa. The lowest strength was achieved by geopolymer samples containing 2% of carbon fiber—6.5 MPa. The surprising result of the research is that the results for the metakaolin matrix and sand for the glass fiber are higher than those for the composites with the addition of carbon fiber roving. By analysis of data from the literature, carbon fiber has higher strength parameters than glass fiber, so composites with its addition should obtain higher properties. The different than expected results can be explained by the better adhesion of the glass fiber to metakaolin. However, further research is required to confirm this hypothesis.

By analyzing the data obtained from the study of fly ash and sand geopolymers, it should also be stated that the strength increases for composites with aramid fibers, while the strength decreases with increasing fiber amount in the matrix. In the case of fly ash composites, reinforcement with glass and carbon fibers did not produce the expected effects, in the form of an increase in bending strength. The values obtained are lower than those for the matrix material. The addition of aramid fibers in the amount of 0.5% increased the strength by almost 2 MPa.

In addition, a comparison of the behavior of materials was carried out on the basis of graphs prepared on the basis of data obtained from the device on which the tests were carried out. The highest values obtained by the selected samples from the given series were selected for comparison.

**Figure 3:**
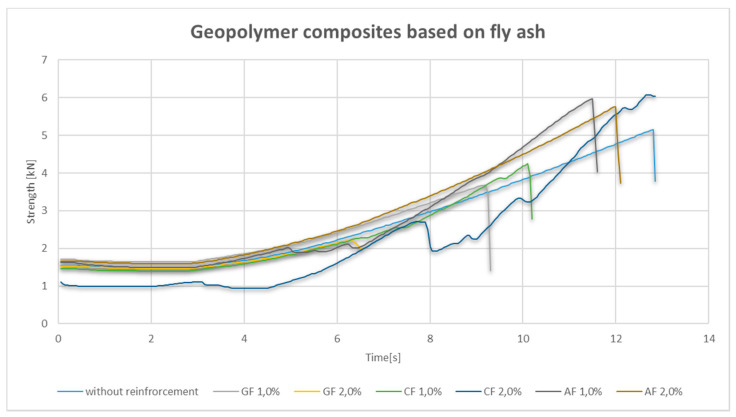
Strength–time graph for the bend test for fly ash composites.

In the case of composites based on fly ash reinforced with 1.0% and 2.0% fibers, the highest value was obtained for 2.0% carbon fiber (CF) (Figure 3). However, the average result for this composite turned out to be lower than for the pure geopolymer matrix material. For most fractures, the fracture indicated in the graph is brittle, with the exception of a composite with 2.0% glass fiber (GF), where a mixed fracture mechanism appears to occur, but the obtained value of the bending strength is very low, and the fact that at this stage the test is carried out on one sample does not allow an unequivocal interpretation of the result (Figure 1). Also interesting is the mechanism seen for the composite containing 1.0% carbon fiber, where local force drops are visible on the graph, such a mechanism may be related to the effect of the fibers that reinforce the composite. The graph for aramid fiber (AF) in the amount of 1.0 vol.% is interesting. There is a clear decrease in strength, followed by slight local drops with a continuous increase in strength. This may be due to a breakdown in the matrix structure and a very slow crack propagation with simultaneous action of aramid reinforcement.

**Figure 4:**
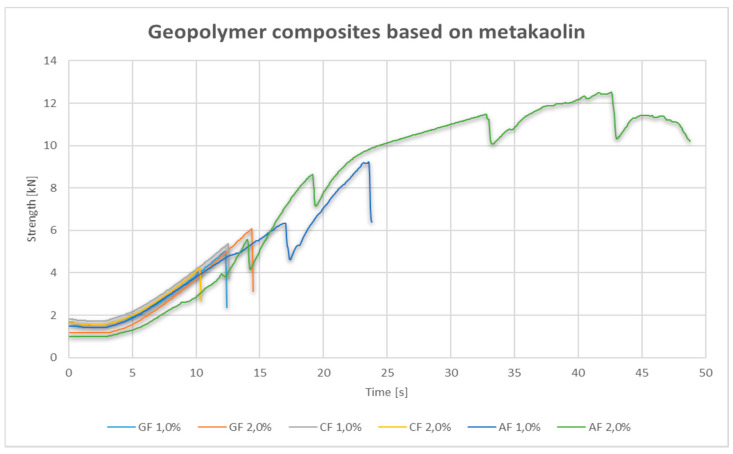
Strength–time graph for the bend test for metakaolin-based composites.

Composites based on metakaolin reinforced with glass and carbon fibers behaved in a similar way during the bending test. The nature of their diagram shows the behavior in a fragile way (Figure 4), which, however, in further analysis, does not fully reflect the behavior of the composite during the destruction mechanism. In the case of metakaolin-based composites, the highest strengths were obtained for composites with 2.0% addition of aramid fiber, they were definitely higher than for composites reinforced with carbon and glass fibers. The average of the measurements for aramid fibers is 40% higher than for glass fiber geopolymers, the strength of which increased with the volume addition of the fibers. An interesting mechanism was observed for 1.0% and 2.0% of aramid fibers, where clear decreases in strength can be observed, followed by its several-fold increase, which is probably due to the impact of the fibers.

**Figure 5:**
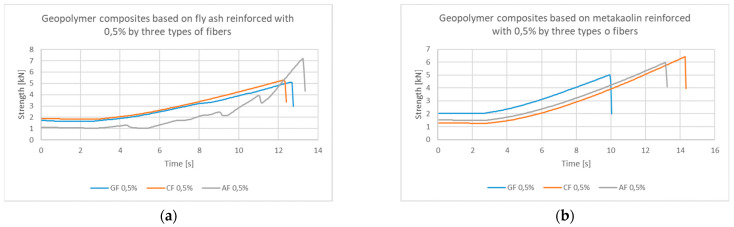
Strength–time comparison of the behavior of composites based on: (**a**) fly ash, (**b**) metakaolin, with the addition of 0.5% roving from glass fiber, carbon fiber, aramid fibers.

Selected charts comparison is as follows:

The comparison was made on the example of 0.5% addition of three types of fibers to the geopolymeric matrix of fly ash and metakaolin. In Figure 5a,b, similar characteristics of the bending mechanisms can be seen. In the case of aramid fiber based on fly ash (FA), the irregular nature of the graph is observed, which may indicate faster cracking of the sample and crack propagation, and finally the strengthening of the material under the influence of fibers, which was not observed in samples based on metakaolin (MK). Interestingly, for 0.5% of the aramid fibers in the metakaolin matrix, a lower strength value was achieved than for the carbon fiber.

**Figure 6:**
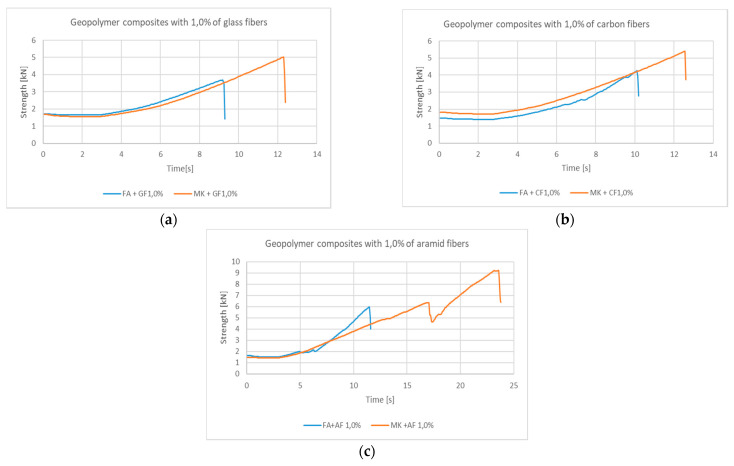
Strength–time comparison of the behavior of composites based on fly ash and metakaolin with the addition of 1.0% roving from: (**a**) glass fiber; (**b**) carbon fiber; (**c**) aramid fibers.

Additionally, for 1.0% fiber addition, the behavior of the fly ash (FA) and metakaolin (MK) composites was compared. It should be noted that with a similar nature of the graphs, the composites based on the metakaolin matrix achieve significantly higher values (Figure 6). However, in the case of the sample with 2.0% aramid fiber content in the metakaolin matrix, after the decrease in force, its further increase occurs, which shows that the fiber is not broken.

**Figure 7:**
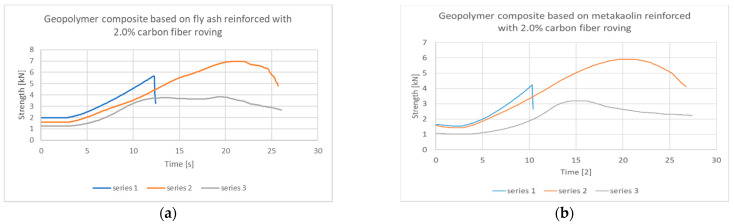
Strength–time composite behavior for a geopolymer matrix based on: (**a**) fly ash with 2.0% carbon fiber roving; (**b**) metakaolin with 2.0% carbon fiber roving.

Interesting results have been achieved as a result of further investigation of the failure mechanism. After completing the test according to the procedure in accordance with the PN-EN 12390-5: 2011 standard: Concrete tests-part 5: The bending strength, photographic documentation of the sample was made, and then a bend test was carried out for samples that were not damaged. For the selected composites, the results turned out to be different than the original assumptions. According to the theory of fracture mechanics, the samples in the first test should obtain the highest value, and in the next test (over time) this value should decrease. In the case of selected composites, this slope may be non-linear, and the sample may “defend itself” against damage (fracture) reaching, in the case of the continuous fracture mechanism, local maxima. This behavior was observed for selected glass fiber reinforced composites and for fly ash composites reinforced with carbon fibers. However, in the case of the tested samples based on both fly ash and metakaolin reinforced with carbon fiber, this mechanism was slightly different. The samples achieved the highest value at the nature of the second bend, with the fracture nature changing from brittle to continuous (Figure 7). The values obtained in the second test, where the composite was decohesive, were higher than in the first test, where only slight cracks appeared, but the material did not lose its cohesion.

**Figure 8:**
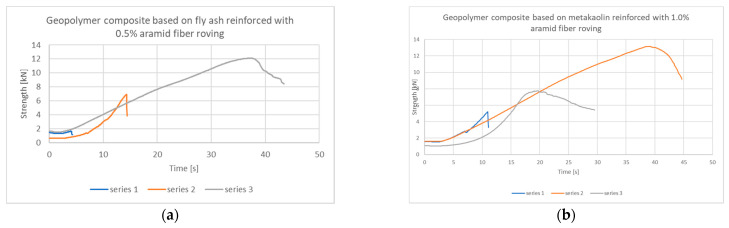
Strength–time composite behavior for a geopolymer matrix based on: (**a**) fly ash with 0.5% aramid fiber roving; (**b**) metakaolin from 1.0% aramid fiber roving, in subsequent series of repetitions.

An interesting example is the 0.5% aramid fiber in the fly ash matrix (Figure 8a), where it was observed that the samples reached the highest value in the third test series. At the same time, in the third deflection, the sample changed the character of the fracture from brittle to continuous. In the case of a metakaolin-based matrix for 1.0% aramid fibers (Figure 8b), the nature of the bending test is similar to that of carbon fiber (Figure 7).

The initial analysis indicated that the nature of the breakthrough did not change, but further tests showed that the nature of the breakthrough for the composite had changed. With the addition of long fibers, the nature of the breakthrough for most composites has changed. A significant change was achieved for carbon fiber composites (both based on a fly ash matrix and metakaolin matrix), as well as for composites based on metakaolin with glass fiber. The ash-glass-fiber composites behaved more similarly to a brittle fracture.

### 3.3. Study of the Fracture Mechanism–Photographic Material

In the case of the analysis of the cracking mechanism, we can most often distinguish three phases:

- Process initiation;
- The spread of cracks in the sample;
- Mechanism of material destruction.

Depending on the material, these phases will have a slightly different character and a different time course.

**Figure 9:**
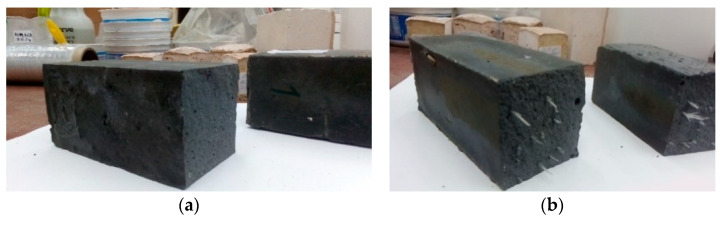
Breakthrough of samples based on fly ash (**a**) without reinforcement; (**b**) with 0.5% addition of glass fiber.

During the bend test, the geopolymeric material without reinforcement behaves in a manner typical of a brittle fracture (Figure 9a). The appearance of microcracks, their propagation, and destruction of the structure occur in a short time interval. The bending test shows a uniform fracture across the entire cross-section of the sample, which is destroyed during the test (most often it breaks in the middle).

A similar mechanism occurs for samples based on a fly ash matrix with a small amount of glass fiber (Figure 9b). Figure 9a,b shows similar brittle material behavior. Fracture in the case of samples was rapid. In the case of glass fibers, the ductile behavior was not observed. The fibers, probably because of the alkaline reaction, lose their tongues and become fragile. This kind of reinforcement was not sufficiently effective for the fly ash–based matrix.

**Figure 10:**
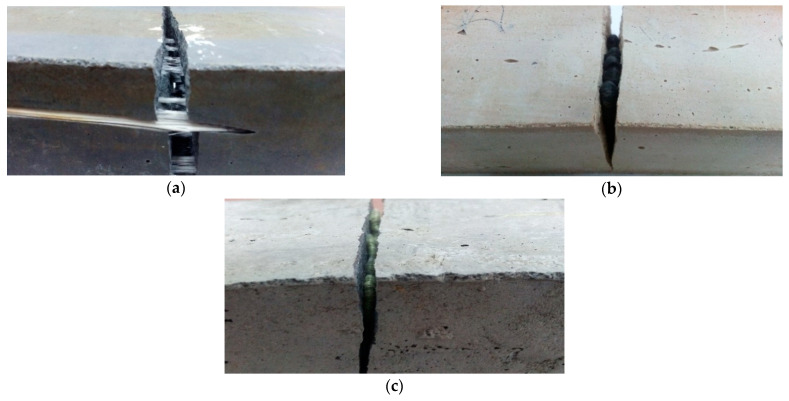
Behavior of different types of fibers in geopolymer composites: (**a**) fly ash composite reinforced with 1.0% glass fiber addition; (**b**) metakaolin-based composite reinforced with 2.0% carbon fiber addition; (**c**) fly ash composite reinforced with 2% addition of aramid fiber.

When more fiber is used, the sample begins to take on a slightly different character and, at the fracture, the resistance of the fiber becomes visible (Figure 10).

Depending on the type of reinforcement, the fibers will behave slightly differently. Depending on their flexibility, they may break with the matrix or retain their elastic character. Their behavior will also depend on their consistency with the matrix material. In case of inconsistency, they can be pulled out of the matrix. In the case of fly ash composites, the nature of the change in fiber behavior was visible. After a short period of “resistance”, it lost coherence (Figure 9a,b). In other cases, the fibers retained their elastic character and did not break. This mechanism was most evident in the case of carbon fiber in a metakaolin-based matrix, where the fiber retained the composite cohesiveness and prevented whole material fracture from propagating (Figure 10b). A similar mechanism was observed for the aramid fiber in the fly ash matrix (Figure 10c).

**Figure 11:**
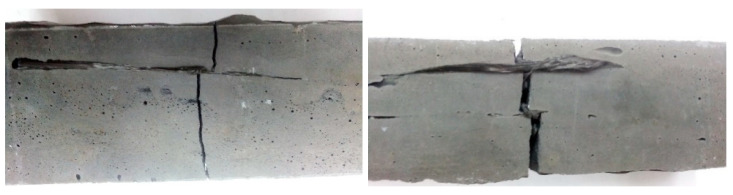
Cracking of fly ash composites reinforced with 1.0% carbon fiber roving additive—a visible mechanism of the reduction of crack propagation through the fibers.

Carbon fiber also effectively counteracted the brittle mechanism in the fly ash matrix, where it reduced the propagation of cracks and/or caused their displacement to other parts of the material along the fiber surface (Figure 11).

**Figure 12:**
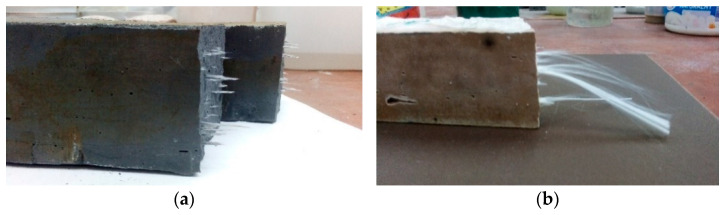
Changing the behavior of glass fibers placed in various geopolymer matrices: (**a**) fly ash-based matrix; (**b**) metakaolin-based matrix.

The research also showed the possibility of changing the behavior of the fiber depending on the matrix, despite its similar nature (Figure 12). Glass fibers behaved differently depending on the matrix. In the fly ash matrix, they became “brittle” and lost their elasticity. The behavior change was likely caused by a reaction with the template material (Figure 12a). In addition, it was observed that the fibers that were on the exposed parts of the samples most exposed to the loss of elasticity were not completely placed in the matrix (Figure 11). In the metakaolin-based geopolymer material, the fibers from the same delivery behaved in a completely different way. During the bending test, they effectively counteracted cracking. After unfolding the composite, they were pulled from one half of the composite, but they did not lose their elasticity (Figure 12b). It has been assumed that such behavior results from the degradation of the protective shell of the jackal fiber, which is aimed at, among other things, protection of the core against the effects of the alkaline environment, which damage the fiber structure; additional microstructural observations were carried out to confirm this hypothesis (presented later in this article).

**Figure 13:**
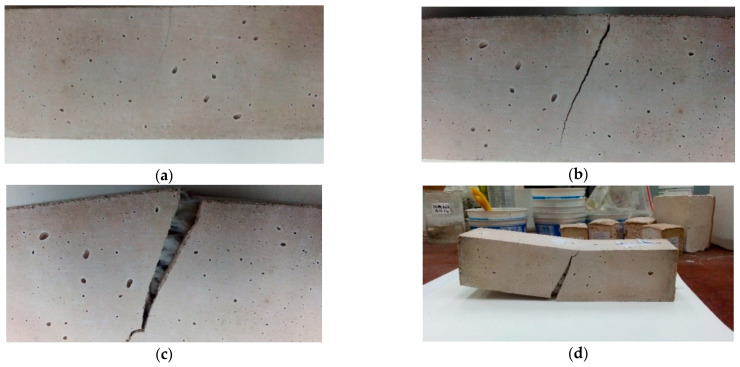
Crack propagation in a geopolymer composite based on metakaolin reinforced with 2.0% glass fiber roving: (**a**) first crack; (**b**) crack after second series of testing; (**c**,**d**) final damage of the sample.

Figure 13 shows the successive stages of material destruction and crack propagation in the sample.

In the first phase, a slight crack appears in the material (Figure 13a), and at this stage the fibers can interact effectively to minimize the spread of cracks in the material. As the force increases further, the fracture deepens (Figure 13b). At this stage, the fibers maintain the integrity of the composite and delay the mechanism of its complete destruction. If the load is continued, the damage to the material increases significantly (Figure 13c) and its cohesion is based on the fibers, with the entire material undergoing significant deformation (Figure 13d).

### 3.4. Evaluation of Fracture Mechanisms-Microstructure Studies

The study of the morphology of the fibers was carried out to determine the degree of their degradation and adhesion to the geopolymer matrix.

**Figure 14:**
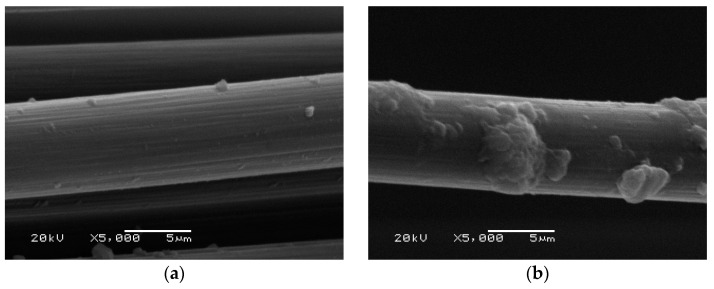
SEM image: Carbon fiber (**a**) as delivered; (**b**) derived from a sample based on a fly ash matrix.

The tests carried out on carbon fibers did not show degradation in the matrix (Figure 14). On the fiber removed from the sample (Figure 14b), the remains of the matrix are visible, but the surface of the fiber itself is not damaged.

**Figure 15:**
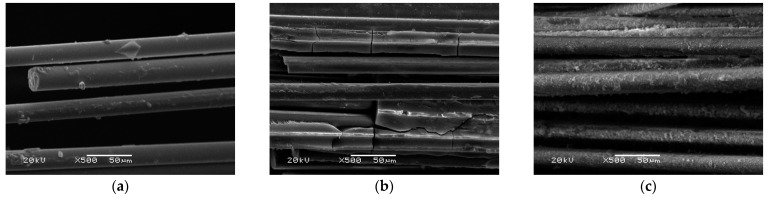
SEM image: Glass fiber: (**a**) as delivered; (**b**) from a sample based on a fly ash matrix; (**c**) from a sample based on a metakaolin matrix.

The tests carried out on glass fibers showed significant damage when they come from a fly ash-based matrix (Figure 15b). Compared to the fibers as delivered (Figure 15a), numerous cracks and delamination of the fiber are visible. For the same fibers that are derived from the metakaolin-based matrix composite (Figure 15c), no damage to the fibers can be seen.

In the case of glass fibers derived from a metakaolin matrix (Figure 15c), they are closely covered with the matrix material, demonstrating a much better cohesion of these fibers with the matrix material than in the case of carbon fibers (Figure 14b), where only small areas of coverage with the matrix material are visible. This phenomenon may explain the high strength properties achieved by these composites in comparison with composites with carbon fibers.

**Figure 16:**
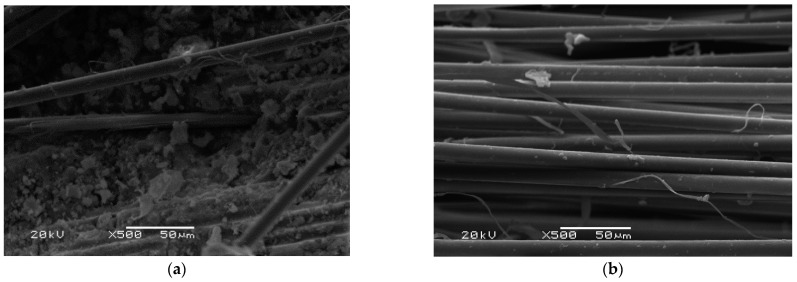
Aramid fiber in the fly ash matrix: (**a**) after the first series of bending tests; (**b**) after the second series.

The SEM photos were taken for a geopolymer composite with aramid fiber 1.0% in a fly ash matrix, for which the result in the second series was 19.3 MPa. After the first destruction of the geopolymer matrix in the flexural strength test, a clear location of the fiber in the matrix can be observed (Figure 16a). On the other hand, in the case of the view of the fibers after the second series (Figure 16b), only small areas with the remnant of the material are observed, which indicates a loss of cohesion with the matrix material.

In the case of aramid fibers (Figure 16) and carbon fibers (Figure 14), the mechanism of destruction of the fiber surface was not as strong as in the case of glass fibers. The aramid fibers retained their ductile nature and work effectively as a reinforcement during the bending test.

## 4. Discussion

The stage of research conducted on the fracture mechanics of composites shows that the proper addition of fibers changes the nature of the fracture from brittle to more ductile. The number of cracks in the material is reduced (in particular, the propagation of microcracks is reduced), as well as their dimensions—the width of the cracks is limited. Overall brittle behavior is suppressed in favor of increased ductility. As a result, damage caused by brittle fracture is minimized and the consistency of the material can be maintained for longer.

Increasing the amount of glass and aramid fibers in the fly ash matrix decreased the strength of geopolymer composites, which may be caused by an increase in the porosity of the material [45]. On the other hand, in the case of materials with a metakaolin-based matrix, this trend is reversed. This may be the result of greater adhesion of said fibers to the matrix material.

As in the case of the analysis [54], it is possible to confirm the positive effect of fibers on the improvement of the strength properties and the critical beginning of cracking, provided that the mixtures of both the matrix material and the reinforcement are properly selected. This increases the adhesiveness of the fibers to the matrix and changes the nature of the material from brittle to ductile, which can be successfully used for further research on the suitability for 3D printing technology.

The important issue in the case of geopolymer materials application is also the limitation of the environmental influence. The technology for producing Portland cement, which is the basic component of concrete, has many disadvantages that burden the natural environment, such as the emission of huge amounts of CO_2_, the consumption of energy or the use of large amounts of natural resources [3,9]. The pace of climate change that Earth’s inhabitants are struggling with is accelerating more and more, and the effects of these changes are noticeable to everyone. An important direction of development is therefore looking for new, more ecological materials, in particular those based on renewable raw materials [4,5,6,7]. One of the promising alternatives may be geopolymerization technologies with a much lower carbon footprint compared to traditional building materials [21]. The provided Life Cycle Analysis (LCA) or environmental assessments for these materials confirmed that products made with the use of geopolymer materials could have a lower environmental influence, but only if the proper manufacturing technique is used and the products are designed taking into account eco-design rules, such as the use of local raw materials [70,71,72]. Moreover, the important issues that could imitate geopolymer applications are the changeable costs of ingredients [32]. The cost effectiveness of particular applications is related to the particular products and technologies. The cost analysis provided in developed countries shows that, with the use of advanced technologies, geopolymers could be more cost effective than traditional concreates in innovative applications [4,70].

## 5. Conclusions

In the article, the structure and mechanical properties in terms of cracking mechanics were presented. The investigation was carried out on geopolymer composites based on fly ash and metakaolin with three types of reinforcement material: glass, carbon, and aramid long fibers. Each of them was added in 0.5%, 1%, and 2% by volume. The obtained results show that:

- The addition of fibers did not significantly affect the density of the materials. The density obtained from the composites was between 1.72 g/cm^3^ for fly ash-based composites with 2% aramid fibers and 1.83 g/cm^3^ for fiberglass-based metakaolin composites.
- Reinforcement with aramid fiber in the amount of 2%, resulted in more than 3 times increase in bonding strength, compared to composites without reinforcement. The lowest value was obtained for fly ash–based geopolymers with 2% glass fiber—5.6 MPa. It was probably caused by fiber degradation in the matrix. The highest bending strength was 18.1 MPa for the metakaolin-based composite with 2.0% aramid fiber admixtures.
- The fracture mechanism in long fiber reinforced geopolymer composites cannot be determined by simple bending tests (in some cases the obtained values will be significantly lower than the actual values leading to material failure); this test will not reflect the actual change in the nature of the fracture from brittle to more continuous. Proper addition of fibers changes the nature of the fracture from brittle to more ductile and reduces the number of cracks in the material.
- Glass fibers (4 times cheaper than carbon and aramid fibers) may be an effective method of reinforcing composites, especially for metakaolin-based geopolymers, and in some cases they may be more effective than carbon fibers. However, their future application requires a more advanced test about reaction fiber matrix in a particular environment, including significant degradation of the fibers in fly ash–based composites.
- An analysis of the morphology of the fibers shows that careful selection of fibers for the matrix is essential, as they can degrade, which is especially important in the case of geopolymer matrices. This mechanism significantly reduces the mechanical properties of the composite.

Geopolymer composites reinforced with fibers can be used effectively in additive technologies, but it is important to pay attention to placing the entire fiber inside the matrix (without contact with the external environment). The further application will also require the development of some standards for geopolymer materials.

## Figures and Tables

**Table 1:** Compositions of used tap water [69].

Indicator	Hue	Turbidity	pH	Specific Electrical Conductivity in 25 °C	Oxidability with KMnO_4_	Fluorides	Chlorides	Ammonium Ion	Nitrites	Nitrates
Unit	mg/dm^3^	NTU	-	μS/cm	mg/dm^3^	mg/dm^3^	mg/dm^3^	mg/dm^3^	mg/dm^3^	mg/dm^3^
Used Tap Water	2	<0.1	7.8	650	<0.7	0.13	29.9	0.022	<0.01	20
WHO Regulations for Drink Water	15	5	not required	not required	not required	1.5	250	1.5	0.5	50
**Sulphates**	**Calcium**	**Magnesium**	**Iron**	**Manganese**	**Copper**	**Chromium**	**Nickel**	**Cadmium**	**Chloroform**	**Water Hardness**
mg/dm^3^	mg/dm^3^	mg/dm^3^	mg/dm^3^	mg/dm^3^	mg/dm^3^	mg/dm^3^	mg/dm^3^	mg/dm^3^	mg/dm^3^	292 μg/dm^3^
32	108	10.2	<0.025	<0.002	<0.003	<0.002	<0.0025	<0.00045	<0.3	2.9 mmol/dm^3^
250	not required	not required	0.3	0.5	2	0.05	0.02	0.005	200	5.8 mval/dm^3^

**Table 2:** Compositions of prepared geopolymer composites.

Sample Series/Number of Samples	Matrix	Reinforcement
S0/2	Fly ash and sand	-
S1/3	Fly ash and sand	Fiberglass 0.5%
S2/1	Fly ash and sand	Fiberglass 1.0%
S3/2	Fly ash and sand	Fiberglass 2.0%
S4/2	Fly ash and sand	Carbon fiber 0.5%
S5/2	Fly ash and sand	Carbon fiber 1.0%
S6/2	Fly ash and sand	Carbon fiber 2.0%
S7/2	Fly ash and sand	Aramid fiber 0.5%
S8/2	Fly ash and sand	Aramid fiber 1.0%
S9/2	Fly ash and sand	Aramid fiber 2.0%
S10/2	Metakaolin and sand	-
S11/2	Metakaolin and sand	Fiberglass 0.5%
S12/2	Metakaolin and sand	Fiberglass 1.0%
S13/2	Metakaolin and sand	Fiberglass 2.0%
S14/2	Metakaolin and sand	Carbon fiber 0.5%
S15/2	Metakaolin and sand	Carbon fiber 1.0%
S16/2	Metakaolin and sand	Carbon fiber 2.0%
S17/2	Metakaolin and sand	Aramid fiber 0.5%
S18/2	Metakaolin and sand	Aramid fiber 1.0%
S19/2	Metakaolin and sand	Aramid fiber 2.0%

## Data Availability

Data sharing is not applicable.
